# Supplementary material for: Causal Relationship between Aging and Anorexia Nervosa: A White-Matter-Microstructure-Mediated Mendelian Randomization Analysis
Source: Biomedicines. 2024 Aug 16;12(8):1874. doi: 10.3390/biomedicines12081874 (PMC11351342; doi:10.3390/biomedicines12081874)
Supplement: Supplementary file 1 [file biomedicines-12-01874-s001.zip › Supplementary Figure/Figure S4/Singe SNP plot of MR analysis for TL to ubm-b-1750.pdf]

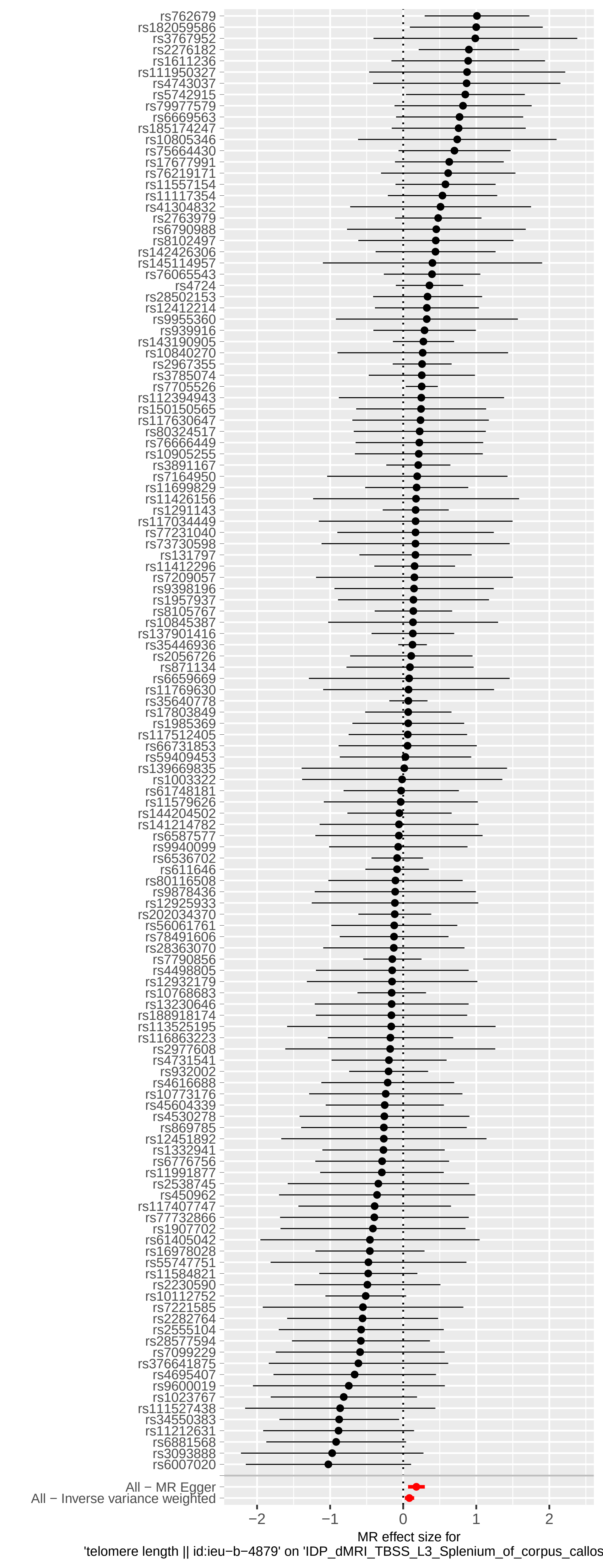
rs762679
rs182059586
rs3767952
rs2276182
rs1611236
rs111950327
rs4743037
rs5742915
rs79977579
rs6669563
rs185174247
rs10805346
rs75664430
rs17677991
rs76219171
rs11557154
rs11117354
rs41304832
rs2763979
rs6790988
rs8102497
rs142426306
rs145114957
rs76065543
rs4724
rs28502153
rs12412214
rs9955360
rs939916
rs143190905
rs10840270
rs2967355
rs3785074
rs7705526
rs112394943
rs150150565
rs117630647
rs80324517
rs76666449
rs10905255
rs3891167
rs7164950
rs11699829
rs11426156
rs1291143
rs117034449
rs77231040
rs73730598
rs131797
rs11412296
rs7209057
rs9398196
rs1957937
rs8105767
rs10845387
rs137901416
rs35446936
rs2056726
rs871134
rs6659669
rs11769630
rs35640778
rs17803849
rs1985369
rs117512405
rs66731853
rs59409453
rs139669835
rs1003322
rs61748181
rs11579626
rs144204502
rs141214782
rs6587577
rs9940099
rs6536702
rs611646
rs80116508
rs9878436
rs12925933
rs202034370
rs56061761
rs78491606
rs28363070
rs7790856
rs4498805
rs12932179
rs10768683
rs13230646
rs188918174
rs113525195
rs116863223
rs2977608
rs4731541
rs932002
rs4616688
rs10773176
rs45604339
rs4530278
rs869785
rs12451892
rs1332941
rs6776756
rs11991877
rs2538745
rs450962
rs117407747
rs77732866
rs1907702
rs61405042
rs16978028
rs55747751
rs11584821
rs2230590
rs10112752
rs7221585
rs2282764
rs2555104
rs28577594
rs7099229
rs376641875
rs4695407
rs9600019
rs1023767
rs111527438
rs34550383
rs11212631
rs6881568
rs3093888
rs6007020
All − MR Egger
All − Inverse variance weighted
−2
−1
0
1
2
MR effect size for
'telomere length || id:ieu−b−4879' on 'IDP_dMRI_TBSS_L3_Splenium_of_corpus_callos
